# Supplementary material for: PEDF Protects Endothelial Barrier Integrity during Acute Myocardial Infarction via 67LR
Source: Int J Mol Sci. 2023 Feb 1;24(3):2787. doi: 10.3390/ijms24032787 (PMC9917376; doi:10.3390/ijms24032787)

Figure1D-ZO-1

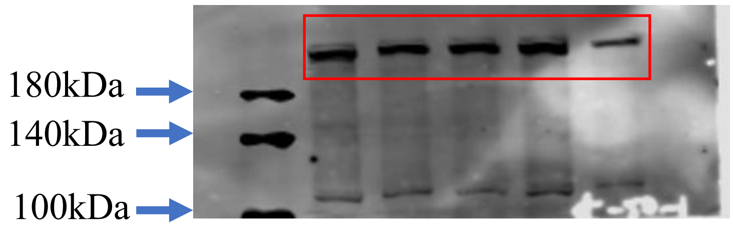

Figure1D-β-Tubulin

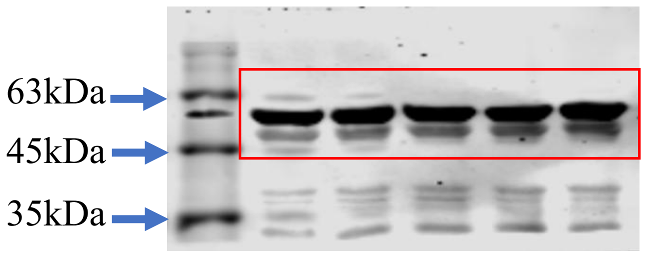

Figure2A-ZO-1

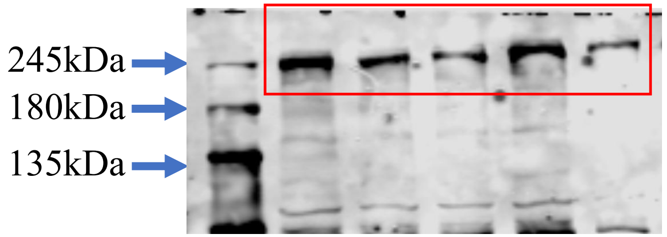

Figure2D-ZO-1

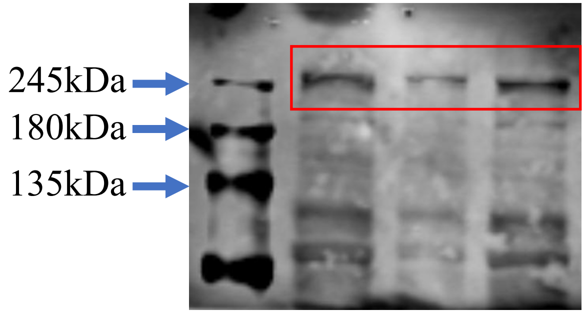

Figure2A-β-Tubulin

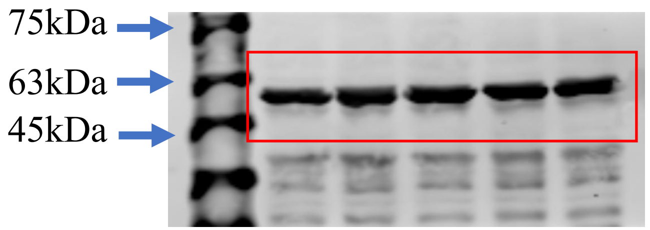

Figure2D-β-Tubulin

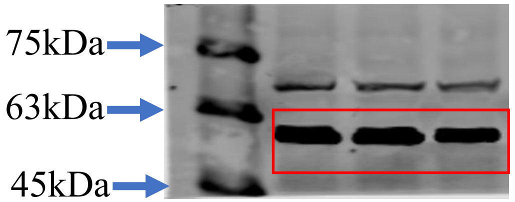

Figure3A-PI3K

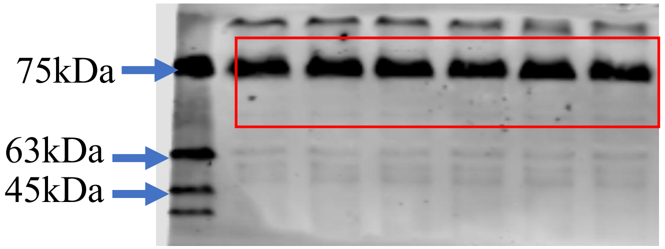

Figure3A-AKT

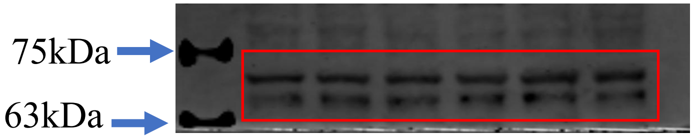

Figure3A-mTor

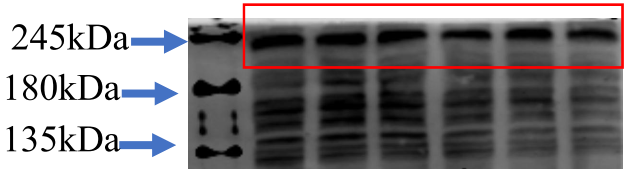

Figure3A-ZO-1

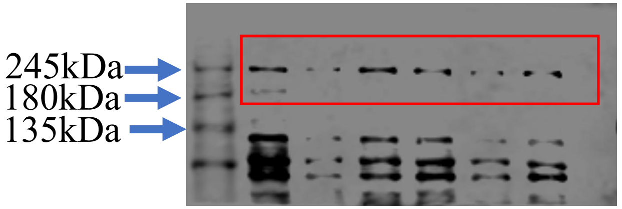

Figure3A-P-PI3K

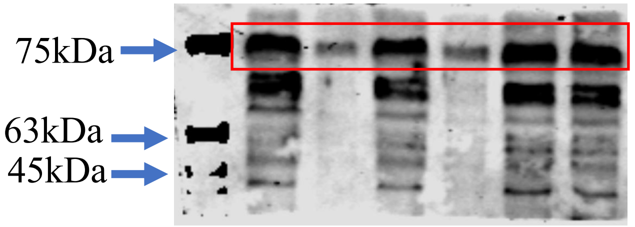

Figure3A-P-AKT

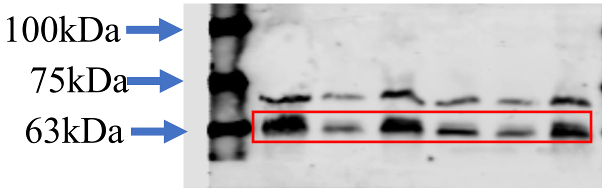

Figure3A-P-mTor

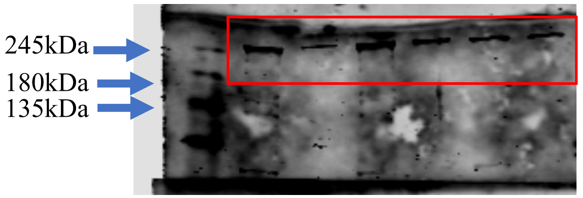

Figure3A-β-Tubulin

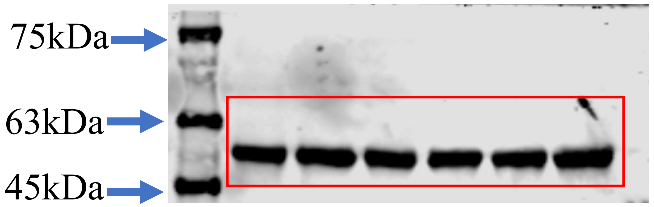

Figure4A-PEDFR

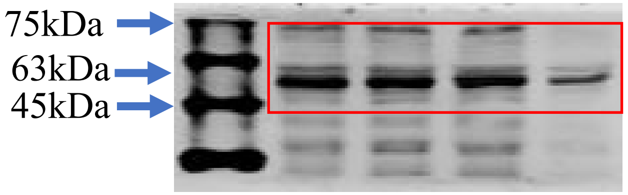

Figure4A-67LR

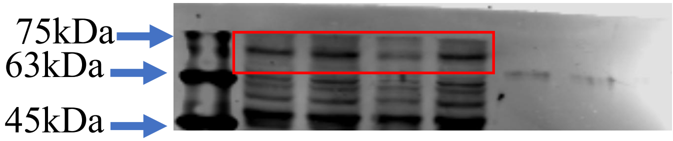

Figure4A-β-Tubulin

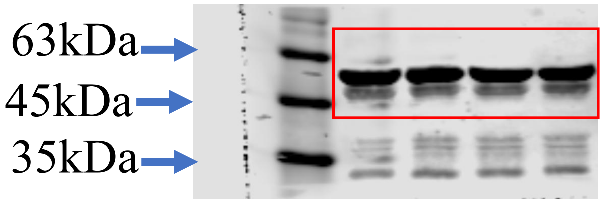

Figure4D-ZO-1

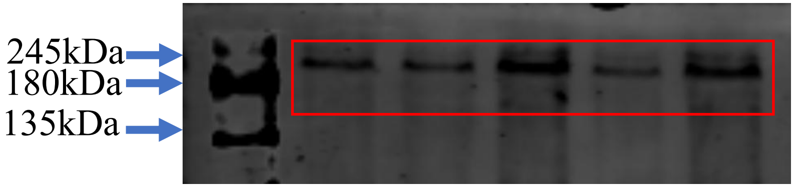

Figure4D-β-Tubulin

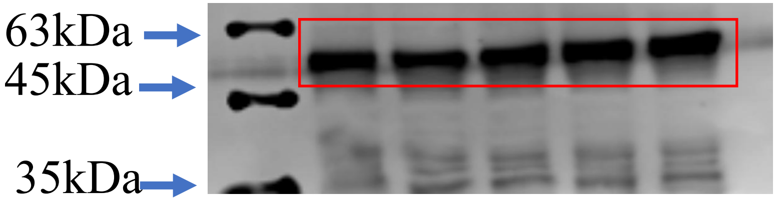

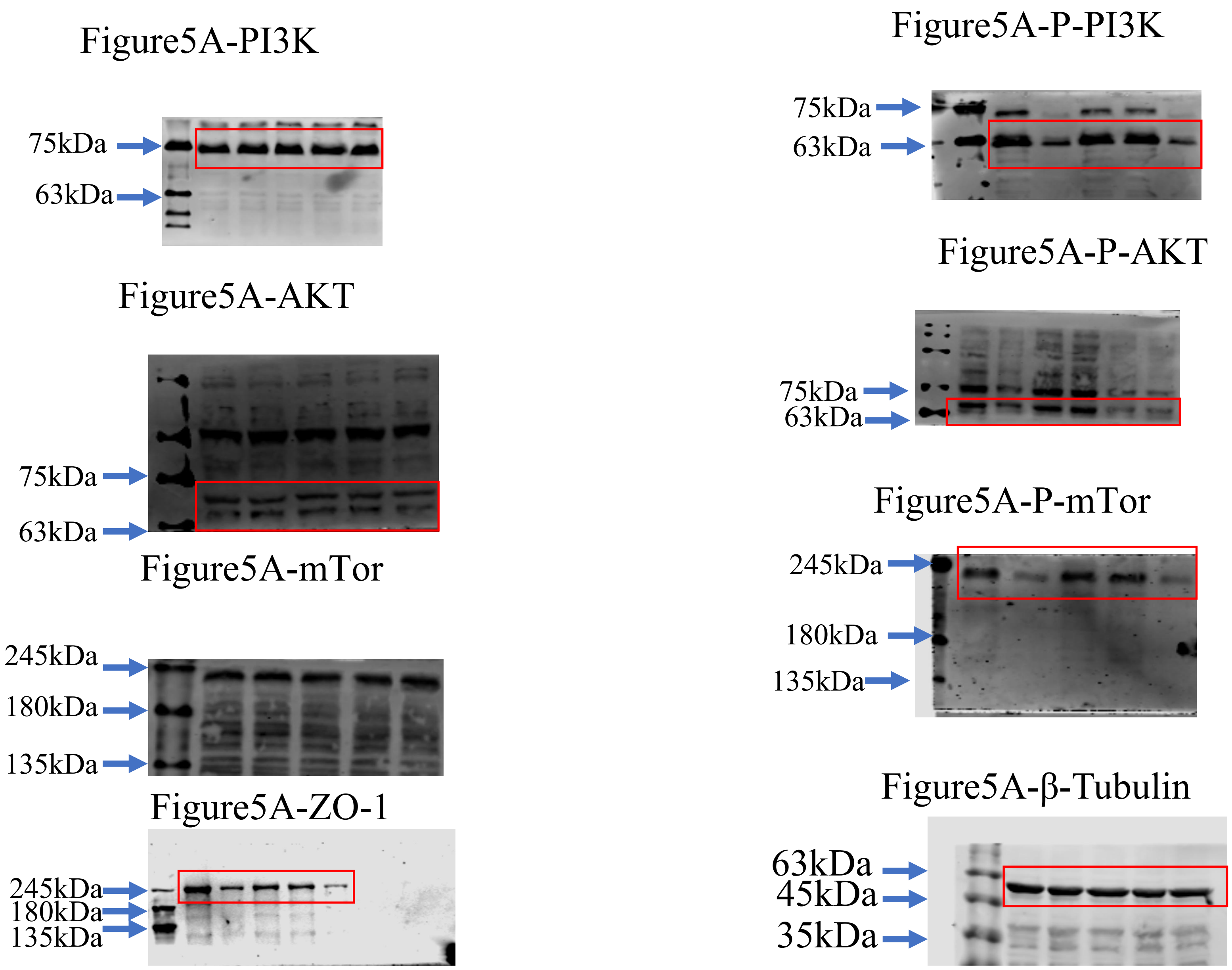
Figure5A-PI3K
75kDa
63kDa
Figure5A-AKT
75kDa
63kDa
Figure5A-mTor
245kDa
180kDa
135kDa
Figure5A-ZO-1
245kDa
180kDa
135kDa
Figure5A-P-PI3K
75kDa
63kDa
Figure5A-P-AKT
75kDa
63kDa
Figure5A-P-mTor
245kDa
180kDa
135kDa
Figure5A-β-Tubulin
63kDa
45kDa
35kDa

FigureS1-PEDF

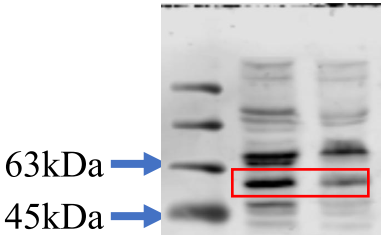

FigureS1-β-Tubulin

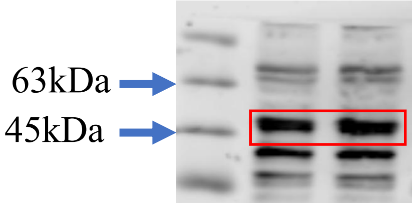

FigureS2-PEDF

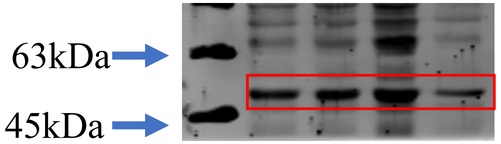

FigureS2-β-Tubulin

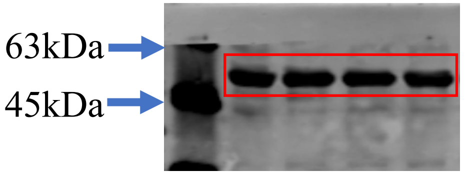

Supplement: Supplementary file 1 [file ijms-24-02787-s001.zip › WB .pdf]
