# Supplementary figures and images for: PEDF Protects Endothelial Barrier Integrity during Acute Myocardial Infarction via 67LR
Source: Int J Mol Sci. 2023 Feb 1;24(3):2787. doi: 10.3390/ijms24032787 (PMC9917376; doi:10.3390/ijms24032787)

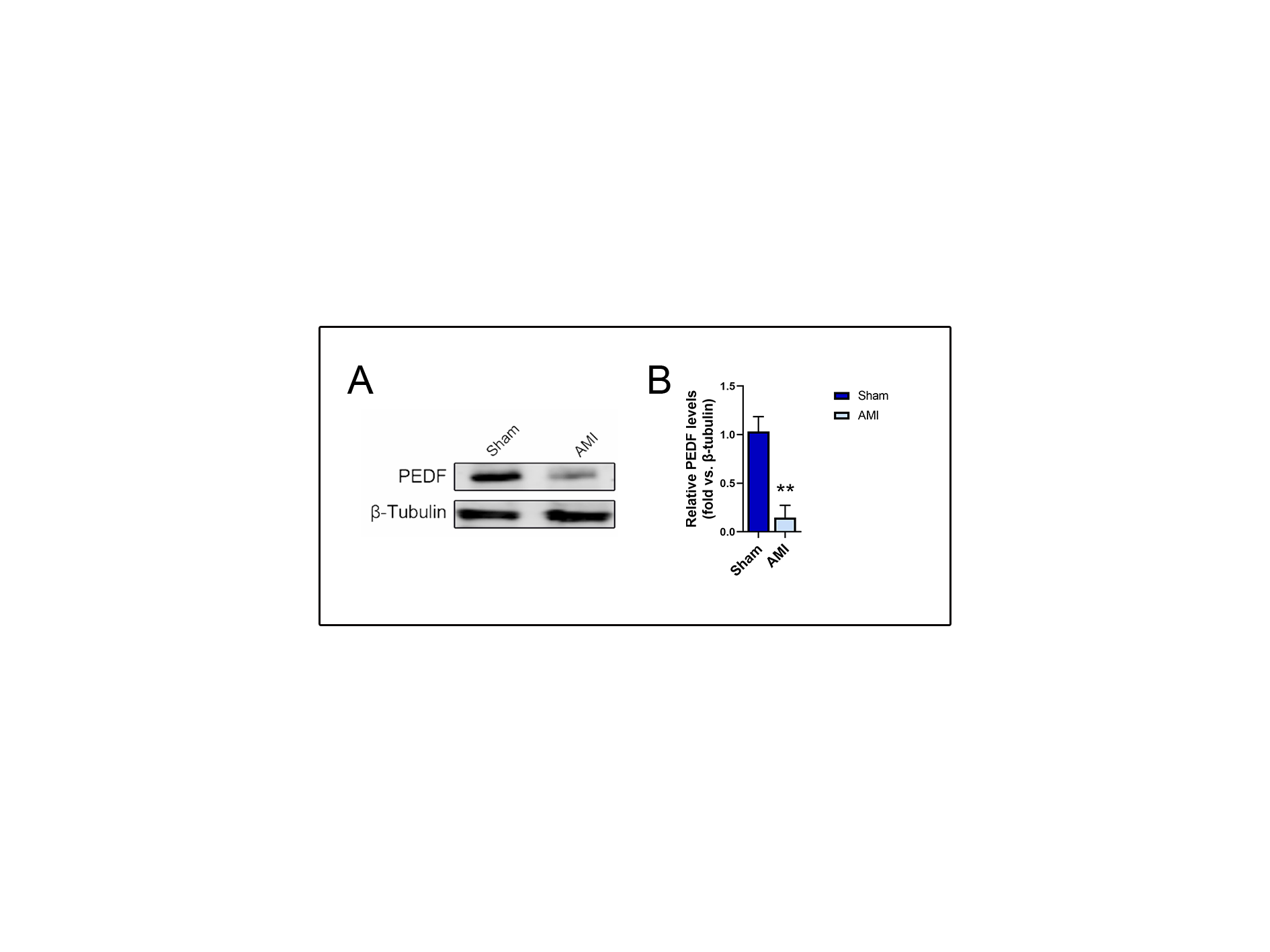

Supplement: Supplementary file 1 [file ijms-24-02787-s001.zip › ijms-2103778-Figure S1.png]

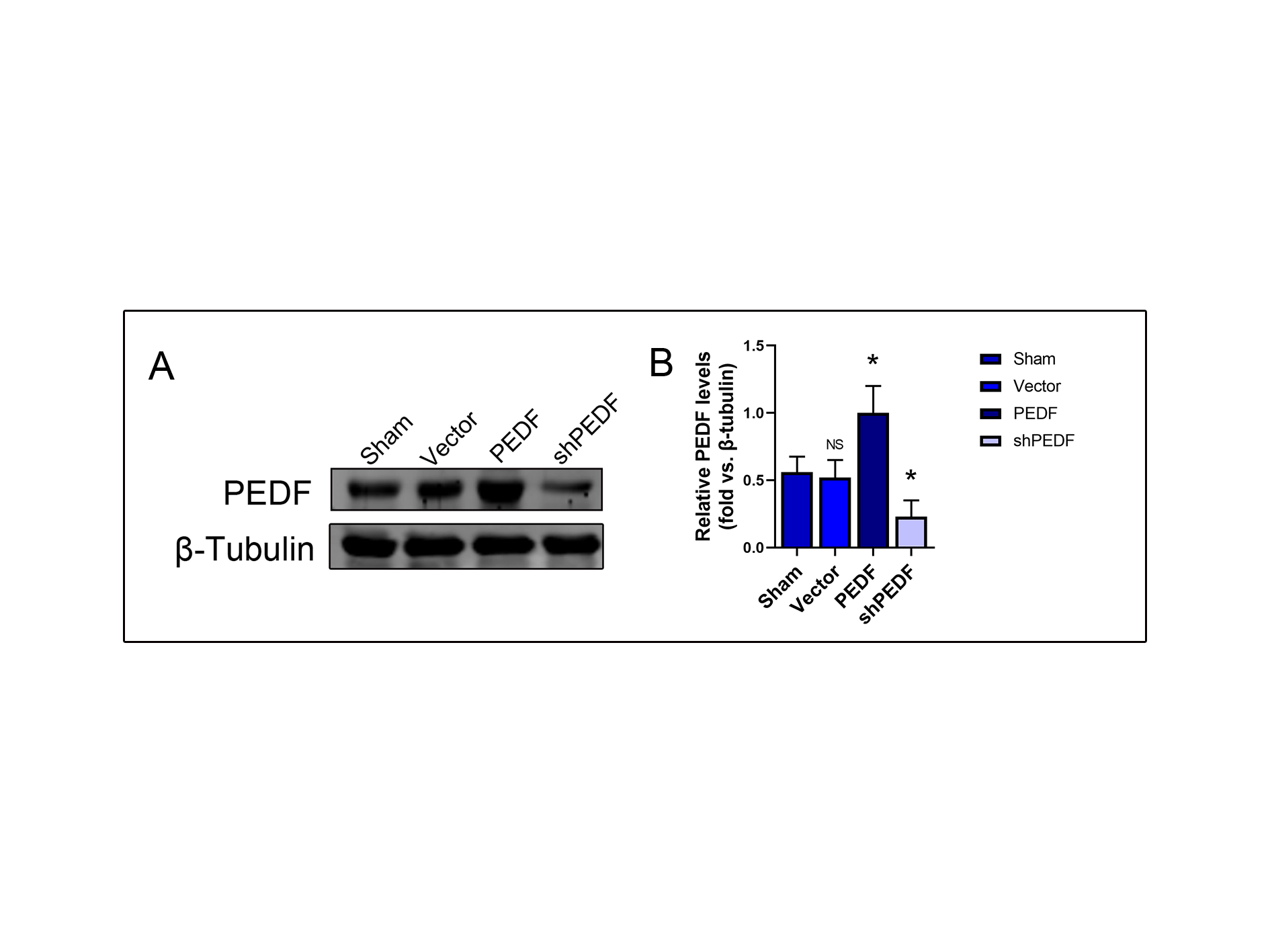

Supplement: Supplementary file 1 [file ijms-24-02787-s001.zip › ijms-2103778-Figure S2.png]

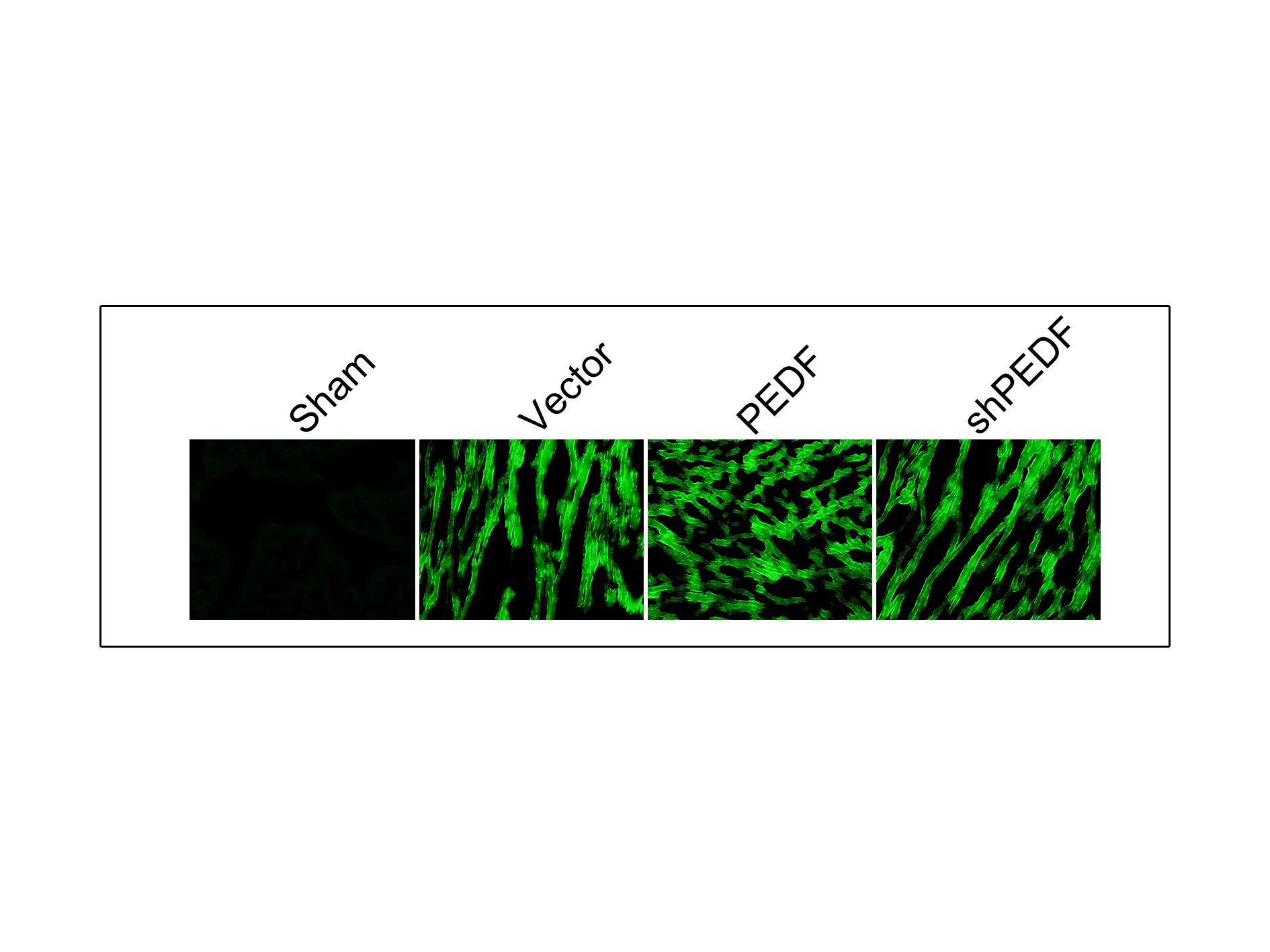

Supplement: Supplementary file 1 [file ijms-24-02787-s001.zip › ijms-2103778-Figure S3.png]
